# Neuronal toll like receptor 9 contributes to complete Freund’s adjuvant-induced inflammatory pain in mice

**DOI:** 10.3389/fnmol.2022.1008203

**Published:** 2022-10-06

**Authors:** Yu Chen, Hui Chen, Xiao-Chen Li, Wen-Li Mi, Yu-Xia Chu, Yan-Qing Wang, Qi-Liang Mao-Ying

**Affiliations:** ^1^Department of Integrative Medicine and Neurobiology, School of Basic Medical Sciences, Shanghai Medical College, Institute of Acupuncture Research, Institutes of Integrative Medicine, Fudan University, Shanghai, China; ^2^Shanghai Key Laboratory of Acupuncture Mechanism and Acupoint Function, Fudan University, Shanghai, China; ^3^State Key Laboratory of Medical Neurobiology and MOE Frontiers Center for Brain Science, Institutes of Brain Science, Fudan University, Shanghai, China

**Keywords:** inflammatory pain, toll-like receptor 9, toll-like receptor 9 antagonist, CpG ODN, spinal cord, neuron

## Abstract

Toll like receptor 9 (TLR9) is a critical sensor for danger-associated molecular patterns (DAMPs) and a crucial marker of non-sterile/sterile inflammation among all TLRs. However, the significance of TLR9 in inflammatory pain remains unclear. Here, we subcutaneously injected Complete Freund’s adjuvant (CFA) into the plantar surface of the hind paw, to established a mouse model of inflammatory pain, and we examined expression and distribution of TLR9 in this model. There was a significant increase of TLR9 mRNA and reduction of mechanical paw withdrawal threshold in mice intraplantar injected with CFA. By contrast, mechanical paw withdrawal threshold significantly increased in mice treated with TLR9 antagonist ODN2088. Furthermore, TLR9 is found predominantly distributed in the neurons by immunofluorescence experiment. Accordingly, neuronal TLR9 downregulation in the spinal cord prevented CFA-induced persistent hyperalgesia. Overall, these findings indicate that neuronal TLR9 in the spinal cord is closely related to CFA-induced inflammatory pain. It provides a potential treatment option for CFA-induced inflammatory pain by applying TLR9 antagonist.

## Introduction

Toll-like receptors (TLRs) are a class of pattern-recognition receptors that are inherited from the germ line and trigger innate immune responses by recognizing pathogen associated molecular patterns (PAMPs) (Akira et al., 2006). Some endogenous ligands such as danger-associated molecular patterns (DAMPs) can also be recognized by TLRs after tissue injury or cellular stress. When activated, TLRs are known to promote the production of a wide variety of inflammatory mediators including cytokines (e.g., TNF-α), chemokines [e.g., monocyte chemotactic protein-1 (MCP-1)], enzymes (e.g., cyclooxygenase-2 and matrix metalloproteinase 9), and other inflammatory mediators (e.g., prostaglandins) (Basbaum et al., 2009; Buchanan et al., 2010; Chen and Nunez, 2010; Lehnardt, 2010; Okun et al., 2011). Therefore, TLRs play a significant part in the pathogenesis of various CNS disorders, including infectious diseases such as spinal cord injury (Pallottie et al., 2018; Li et al., 2019), Parkinson’s disease (Maatouk et al., 2018), neuropathic pain (Liu et al., 2016), pruritus (Liu et al., 2010), stroke (Caso et al., 2007), and multiple sclerosis (Prinz et al., 2006). Proinflammatory central immune signaling pathway plays an important role in the induction and maintenance of heightened pain condition (Nicotra et al., 2012).

TLRs have been involved in the pain process in a growing number of studies, including cancer induced bone pain (Li et al., 2013), neuropathic pain (Wang et al., 2013; Liu et al., 2016, 2017; Zhang et al., 2018) and inflammatory pain (Guan et al., 2016; Liu et al., 2016; Zhang and Deng, 2017; Sun et al., 2018). TLR9 is a critical sensor for DAMPs and a crucial marker of non-sterile/sterile inflammation among all TLRs. TLR9, in particular, is of special role because it localizes in endolysosomes and has distinct ligand, the unmethylated CpG motifs of bacterial DNA (Krieg et al., 1998; Hemmi et al., 2000; Park et al., 2008). Cytidine-phosphateguanosine oligodeoxynucleotide 2088 (CpG ODN 2088) has been used as TLR9 antagonist in numerous studies (David et al., 2013, 2014; Qin et al., 2014; Acioglu et al., 2016). According to the literature, CpG ODN 2088 reduces injury-induced thermal hypersensitivity by inhibiting inflammatory response and TNF-α expression, suggesting that TLR9 may play potential a role in SCI-induced pain (David et al., 2013). Although research efforts have been made on other TLRs, such as TLR4 in inflammatory pain (Zhao et al., 2015), the significance of TLR9 in inflammatory pain is yet unknown.

The current study aims to investigate the role of neuronal TLR9 in the spinal dorsal horn in CFA-induced inflammatory pain. CFA treatment induced upregulation of TLR9 mRNA in the spinal cord, and inhibition of TLR9 by TLR9 antagonist significantly relieved CFA-induced mechanical allodynia. The role of neuronal TLR9 in CFA-induced inflammatory pain was observed by an adeno associated virus (AAV) virus with a neuronal specific promoter SYN delivering TLR9 shRNA to downregulate neuronal TLR9. The present study revealed that neuronal TLR9 might serve as a promising therapeutic approach for inflammatory pain treatment.

## Materials and methods

### Experimental animals

Adult male C57BL/6 mice were purchased from Shanghai SLAC Laboratory Animal Co., Ltd., China. Mice were reared under a 12 h light and dark cycle with free access to food and water. Before experimental operation, mice were acclimated for at least 1 week after being received. The virus injection was applied on mice at the age of 5–6 weeks, while other experiments were performed on mice at the age of 8–10 weeks. This study was approved by the local ethical committee at School of Basic Medical Sciences, Fudan University, People’s Republic of China (Agreement No. 20140226-087). All procedures covered in the study were in accordance with the National Institutes of Health Guide for the Care and Use of Laboratory Animals and the Ethical Issues of the International Association for the Study of Pain. As for behavioral test, prior to formal test, mice were habituated in the testing environment without any stimulation 1 h per day for 2 consecutive days and the experimenters were blinded to the treatment.

### Establishment of complete Freund’s adjuvant-induced inflammatory pain model and intrathecal injection

According to previous study (Ghasemlou et al., 2015), 20 μl of CFA (Sigma, F5881-10ML) was delivered into the plantar surface of hind paw in mice via subcutaneous injection to induce inflammatory-pain related response. The control group was injected with the same volume of normal saline.

Intrathecal injection of ODN 2088 (InvivoGen, tlrl-2088) or ODN 2088 control (InvivoGen, tlrl-2088c-1) (150 ng/g) (David et al., 2014) started on the 5th and 14th day after the CFA injection. A 30 1/2 gauge needle connected with a 10 μl Hamilton syringe was inserted into the intervertebral space between the fifth and sixth lumbar as previously described (Hu et al., 2018). The instant mechanical allodynia was measured every 1 h. ODN 2088 was dissolved in double distilled water. Mice were assigned to the experimental groups at random, and they were anesthetized with isoflurane (1.0 L/min at a concentration of 3.0% in oxygen).

### Real-time PCR

Total RNA of the samples from L4-L6 spinal dorsal horn was isolated with RNSiso Plus (Takara, 9109) following manufacturer’s instructions and then measured with Nanodrop (Thermo). The sequences of primers for each target mRNA are as follows (Acioglu et al., 2016): GAPDH, forward: 5′-AAA TGG TGA AGG TCG GTG TG-3′, reverse: 5′-AGG TCA ATG AAG GGG TCG TT-3′, TLR9, forward: 5′-GCGGCAGCATCCTGCTCCAA-3′, reverse: 5′-GGGGGCTAAGGCCAGTGGGT-3′;. All primers used for PCR analysis are synthesized by Sangon Biotech (Shanghai) Co., Ltd. Reverse transcription was conducted by employing PrimeScript™ RT reagent Kit with gDNA Eraser (Takara, RR047A) and SYBR Premix Ex Taq™ II (Takara, RR820A).

### Behavioral tests

#### Up-and-down method

According to previous research (Hu et al., 2018), a series of von Frey hairs (0.02, 0.04, 0.07, 0.16, 0.4, 0.6, 1.0, and 1.4 g) (Stoelting, Wood Dale, Illinois, USA) were applied to measure mechanical paw withdrawal threshold. In short, mice were acclimated in a plexiglass box separately for 30 min. A von Frey hair was accordingly employed and held for about 3 s. There was a 10-min interval between von Frey hair applications. For each test, 0.16 g von Frey hair was chosen as the first application, and the hair force was then decreased or increased according to the reaction. A rapid withdrawal of the hind paw was defined as positive reaction. There are five more stimuli during the test following the first shift in reaction occurred. Final scores were converted to a 50% von Frey threshold using the Dixon up-and-down paradigm (Dixon, 1980).

#### Hargreave’s test and hot plate assay

Thermal hyperalgesia was measured according to our previous study (Mao-Ying et al., 2006). In brief, mice were put into a clear plastic chamber on an elevated glass pane. Radiant heat was then applied to the plantar surface of the hind paw until the mouse lifted its paw. The cut-off time was set to 20 s so as to avoid damage of tissue.

As for hot plate, according to our previous study (Mao-Ying et al., 2006), the temperature was set to 52°C. Withdrawal latency began when the mouse was placed on the plate and terminated when either a rapid withdrawal or paw flinching was observed. Likewise, the cut-off time was set to 20 s so as to avoid damage of tissue. As for the test, there were three replicates in each mouse with enough interval between applications and calculated by the mean value.

#### Open field test replications

Open field test (OFT) was applied to identify whether AAV injection would influence mice’s locomotive ability. The open box device contains a 50 × 50 cm white floor with an opaque wall (40 cm height). It was cleaned up with 5% aqueous acetic acid before each trial. The performance of mice in the open field was recorded with a camera. Finally, the horizontal movement and the vertical movement (two front paws in the air or the mice climbing the wall) of the mice within 5 min were analyzed by the OFT video analysis system, as described previously (Wang et al., 2019).

#### Rotarod test

To evaluate the mice’s acquisition of skillful motor behavior, a rotarod machine with an automatic timer and a falling sensor (ENV-575M, Med Associates, USA) was used. Before test sessions, mice were trained on the static drum for 5 min (two sessions per day) at a relatively slow speed (5 rpm/min) for 2 consecutive days. To test the accelerating rotarod, the rod speed was set to accelerate from 4 to 40 rpm in 300 s. The falling latency was recorded automatically by photocells. As for the test, there were three replicates in each mouse with at least 30-min intervals between applications, and the mean value of falling latency was calculated.

### Intraspinal adeno associated virus delivery

According to our previous study (Ma et al., 2021), mice aged 5–6 weeks received virus delivery 3 weeks before the CFA injection. Mice were deeply anesthetized and placed on a stereotaxic frame. The spine was fixed with the help of two spinal adaptors to fully expose the L4-L5 lumbar space. A glass microelectrode for Nanoliter (with 1.14 mm of outer diameter, 0.53 mm of inner diameter) was applied to insert into the spinal dorsal horn intervertebral space at a depth of 200–300 μm. A motorized perfusion system (Nanoliter 2010, World Precision Instruments) was used to control the rate of virus delivery (60 nl/min). Delivery finished, and the glass microelectrode was then left in spinal dorsal horn for 5 min. With wounds sutured, mice were placed on a heating pad to support recovery. AAV virus vectors employed in our study were purchased from OBiO Technology (Shanghai, China).

### Transfection with TLR9-GFP plasmid and western blot

The plasmid of TLR9 shRNA was designed by OBiO Technology (Shanghai, China). The micro30 shRNA (Tlr9) sequence was cloned into the *Hin*dIII(1494) and *Age*I(1503) sites of the adeno-associated virus vector AOV062 pAAV-SYN-MCS-EGFP-3FLAG. Neuroblastoma cells SY5Y cells were provided by the Shanghai Institute of Cell Biology, Chinese Academy of Sciences (Shanghai, China), according to previous study from our lab (Cui et al., 2020). Dulbecco’s modified Eagles medium (Gibco, USA) containing 10% fetal bovine serum were used to cultivate SY5Y cells. The cells were stored in the cell incubator, with temperature of 37°C and CO_2_ of 5% concentration. Following the manufacturer’s instructions, SY5Y cells were harvested and transfected with 1 μg of cDNA coding for TLR9-GFP with Lipofectamine 3000 (Invitrogen). The cells were lysed using RIPA Lysis Buffer (100 μl/g, Beyotime Biotechology, P0013B). Applying SDS-PAGE, samples were separated with 10% acrylamide gels and then transferred onto polyvinylidene fluoride membranes. The membranes were incubated overnight with TLR9 (Abcam, ab37154) or β-actin (ProteinTech, HRP-60008) primary antibody at 4°C on a shaker table. On the following day, they were washed with TBST. After that, the membranes were incubated with corresponding HRP-conjugated secondary antibody for 2 h at room temperature. The target bands were detected with ImageQuant LAS4000 miniimage analyzer (GE Healthcare, Buckinghamshire, UK) and the gray value of target bands was analyzed by ImageJ software (version 1.47).

### Immunofluorescence

Mice were anesthetized with 10% chloral hydrate. Then they were transcardially perfused with saline and perfused with 4% formaldehyde. L4-L6 segment of the mice spinal cord was soaked in 4% formaldehyde for 4 h, and subsequently in 20 and 30% sucrose. The 30 μm-thick sections were incubated in Superblock Buffer (Thermo, 37580) for 1 h at room temperature, reacted with mouse anti-NeuN (Millipore) or anti-TLR9 (Abcam, ab37154) overnight at 4°C, and then reacted with Alexa-594-conjugated donkey anti-mouse IgG secondary antibodies (Invitrogen) for 2 h at room temperature. After three washes with PBST, the sections were attached using mounting media containing DAPI and were analyzed by confocal microscope (FV1000, Olympus). The mean gray value and the cell amount were analyzed by ImageJ. The Pearson’s R value was measured by the coloc 2 function of ImageJ.

### Statistical analysis

All data analysis was performed through the use of the Prism7 software packages and IBM SPSS 22.0. All data are presented as mean ± standard error of the mean (SEM). A *t*-test was used for direct comparisons between the two groups. Significant differences among groups were checked by two-way ANOVA. Repeated-measures ANOVA was applied to compare the differences between TLR9 shRNA group and the control group. Significance was assumed when *p* < 0.05.

## Results

### Toll like receptor 9, probably neuronal toll like receptor 9, increased in complete Freund’s adjuvant-induced inflammatory pain in mice

**FIGURE 1:**
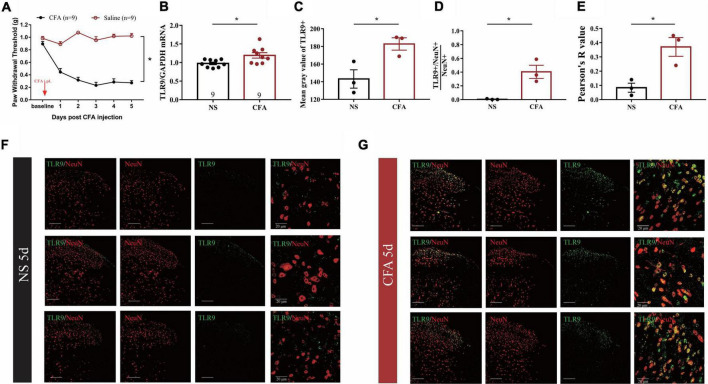
**(A)** The mechanical threshold decreased after CFA injection. The statistical method is repeated measures ANOVA. *p < 0.05. **(B)** Real-time PCR analysis showed that TLR9 increased in inflammatory pain. Results are normalized to GAPDH. Values are represented as mean ± SEM. *p < 0.05. Student’s *t*-test was used for comparisons. **(C)** The mean gray value of TLR9 immunostaining from **(F**,**G)** between NS- and CFA-treated group. Values are represented as mean ± SEM. *p < 0.05. **(D)** The proportion of TLR9 + /NeuN + colocalization between NS- and CFA-treated group. The numbers of cells were counted by ImageJ. Values are represented as mean ± SEM. *p < 0.05. **(E)** The Pearson’s R value of TLR9 and NeuN represented the correlation between NS- and CFA-treated group. The value was measured by coloc 2 in ImageJ. **(F,G)** The immunofluorescence of spinal cord form NS- and CFA-treated group, respectively. Double immunostaining of TLR9 (green) with NeuN (a neuronal marker, red). Scale bars = 100 μm.

We initially established a mouse model of CFA-induced inflammatory pain to observe whether spinal TLR9 was involved in inflammatory pain. In accordance with the previous report (Gao et al., 2018), a significant decline of mechanical paw withdrawal threshold can be observed in CFA-treated mice as compared with normalsaline (NS) treated mice (Figure 1A). Moreover, the results showed that the expression of TLR9 mRNA was increased (*p < 0.05, Figure 1B) in the L4-L6 lumbar segment of the spinal dorsal horn on the 5th day in CFA-treated mice, compared with normal saline-treated mice. Moreover, we measured TLR9 protein levels in NS- and CFA-treated mice by immunofluorescence (Figures 1F,G). The mean gray value of TLR9 increased on the 5th day after the CFA injection (Figure 1C). The proportion of TLR9 + /NeuN + (neuronal nuclei, a neuronal marker) in NeuN + cell was raised in CFA-treated mice compared with NS-treated mice, indicating that the increased TLR9 in CFA-treated mice mainly occurred in neurons (Figure 1D). Further, the Pearson correlation coefficient (Pearson’s *R* value) was higher in CFA-treated mice on the 5th day than that in NS-treated mice, indicating that the positive linear relationship between TLR9 and NeuN was stronger on the 5th day after the CFA injection (Figure 1E). These results showed that CFA-induced inflammation might result in an increase of TLR9 level, especially in neurons.

### Distribution of toll like receptor 9 in the spinal cord of complete Freund’s adjuvant -induced inflammatory mice

**FIGURE 2:**
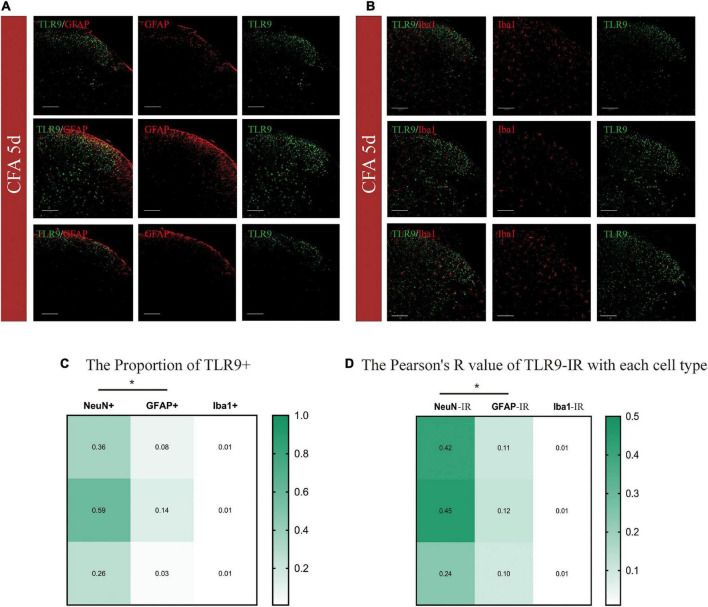
**(A,B)** Cellular distributions of TLR9 in the spinal cords of CFA mice. Double immunostaining of TLR9 (green) with GFAP (an astrocytic marker, red), and Iba-1 (a microglial marker, red) in the spinal dorsal horn. Scale bars = 100 μm. **(C)** The heatmap showed TLR9 + /NeuN + proportion was generally higher than TLR9 + with GFAP + or Iba1 + with proportions of colocalization color-coded in the scale shown. *p < 0.05. Student’s *t*-test was used for comparisons. **(D)** The heatmap showed the Pearson’s *R*-value of TLR9 with each cell type with values color-coded in the scale shown. *p < 0.05.

TLR9 was then doubly immunostained with GFAP (glial fibrillary acidic protein, an astrocytic marker), and Iba-1 (ionized calcium-binding adapter molecule 1, a microglial marker) (Figures 2A,B). According to the color code of the heatmap, the proportion of colocalization between TLR9 + and NeuN + was generally greater than that between GFAP + and Iba1 + (Figure 2C). Moreover, we adopted the Pearson correlation coefficient (Pearson’s R value) to analyze the specific relevance on individual variables (Figure 2D). The Pearson’s R values were 0.42, 0.45, and 0.24 between TLR9 immunoreactivity (IR) and NeuN-IR, representing the positive linear specific relevance (R = [0, 1]) of TLR9 to NeuN. The Pearson’s *R* values of TLR9-IR and NeuN-IR were generally higher than those of GFAP-IR and Iba1-IR. These indicated that in CFA-treated mice, the positive linear specific relevance of TLR9-IR to NeuN-IR were stronger than TLR9-IR to GFAP-IR or Iba-1-IR. TLR9-IR was mainly colocalized with NeuN-IR, suggesting that the alteration of TLR9 was predominantly distributed in neuron in CFA-induced inflammation.

### Toll like receptor 9 was involved in complete Freund’s adjuvant -induced inflammatory pain

**FIGURE 3:**
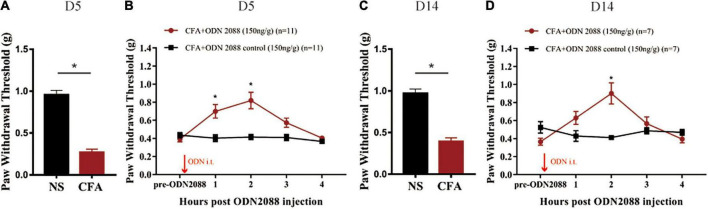
**(A)** The von Frey test showed that the mechanical paw withdrawal threshold decreased at the 5th day after CFA injection. Values are represented as mean ± SEM. *p < 0.05. Student’s *t*-test was used for comparisons. **(B)** TLR9 inhibition raised mechanical paw withdrawal threshold 2 h after the ODN2088 injection at D5. **(C)** The von Frey test showed that the mechanical paw withdrawal threshold decreased at the 14th day after CFA injection. Values are represented as mean ± SEM. *p < 0.05. Student’s *t-*test was used for comparisons. **(D)** TLR9 inhibition raised mechanical paw withdrawal threshold 2 h after the antagomir injection at D14. The arrows indicated the moment of the ODN 2088 or control administrations.

According to Figure 3A, CFA-treated mice exhibited a significant decrease of mechanical paw withdrawal threshold as compared with NS-treated mice on the 5th day after the CFA injection (*p < 0.05). Functional inhibition of TLR9 by intrathecal injection of TLR9 antagonist ODN2088 (150 ng/g body weight; David et al., 2014) significantly increased mechanical paw withdrawal threshold 2 h after the ODN2088 injection (*p < 0.05, Figure 3B), compared to the group with ODN2088 control injection.

Moreover, to evaluate the involvement of TLR9 in long-term inflammation, we also functionally inhibited TLR9 by intrathecally injecting TLR9 antagonist ODN2088 on the 14th day after the CFA injection. CFA-treated mice exhibited a significant decrease of mechanical paw withdrawal threshold as compared with NS-treated mice on the 14th day (*p < 0.05, Figure 3C). TLR9 antagonist ODN2088 significantly increased mechanical paw withdrawal threshold 2 h after the ODN2088 injection (*p < 0.05, Figure 3D), which was alike to the results on day 5.

### Neuronal toll like receptor 9 in the spinal cord contributed to complete Freund’s adjuvant-induced inflammatory pain

**FIGURE 4:**
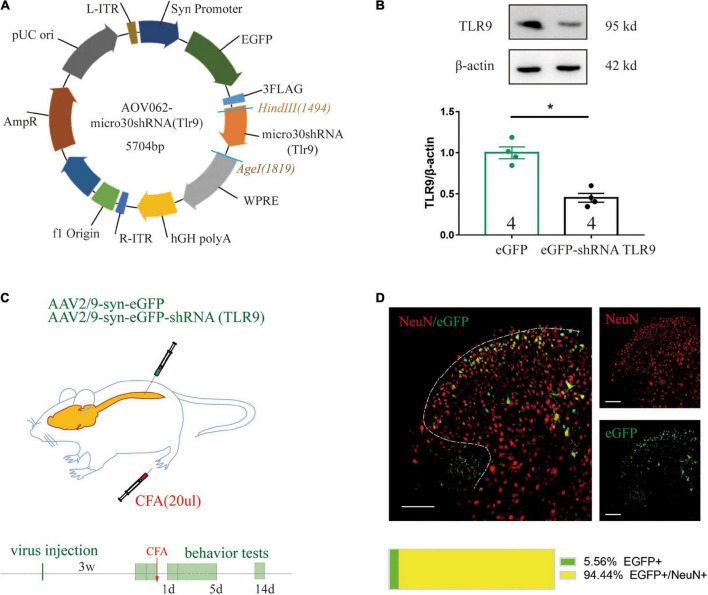
**(A)** The construction of a recombinant plasmid containing shRNA TLR9. **(B)** Western blot analysis showed that TLR9 protein expression was downregulated in SY5Y transfected with TLR9-GFP plasmid. Results are normalized to β-actin and shown as ratios to eGFP control group. Values are represented as mean ± SEM. *p < 0.05 vs. eGFP control group. Student’s *t-*test had been utilized to compare. **(C)** The neuron-specific promoter SYN was used in an experiment to suppress spinal cord neuronal TLR9 expression. **(D)** Representative images showed NeuN labeling (red) and eGFP-tagged (green) AAV-eGFP-shRNA TLR9 in the spinal cord. Scale bar, 100 μm. The percentage of the colocalization was displayed below.

For the purpose of achieving downregulation of TLR9 *in vivo* by AAV, we first constructed a plasmid with effective downregulation of TLR9 in SY5Y cells, according to the previous study from our lab (Cui et al., 2020). The micro30 shRNA (Tlr9) sequence was cloned into the *Hin*dIII(1494) and *Age*I(1503) sites of the AAV vector AOV062 pAAV-SYN-MCS-EGFP-3FLAG (Figure 4A). Western blot analysis showed significant knock-down effects of this plasmid (Figure 4B). Taking SYN as a promoter of mature neurons, the plasmid was packaged into the AAV2/9 vector, which can specifically transfect mature neurons. To assess whether neuronal TLR9 in the spinal cord was involved in CFA-induced persistent hyperalgesia, the virus with the neuronal specific promoter SYN delivering TLR9 shRNA was injected into the L4-L5 lumbar segment (Figure 4C). The EGFP image (green) was highly colocalized with neurons (red) in the dorsal spinal cord (Figure 4D).

**FIGURE 5:**
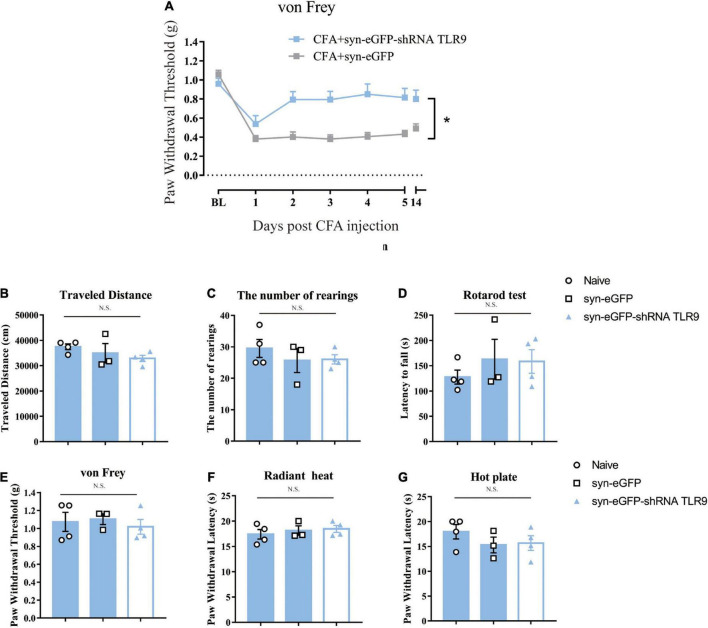
**(A)** Downregulation of neuronal TLR9 in the spinal cord increased the mechanical paw withdrawal threshold in CFA mice. Data are expressed as means ± SEM. Repeated measurements ANOVA is the statistical approach used. **(B–G)** The locomotive ability (open field, rotarod test), the mechanical sensitivity (von Frey) and thermal (radiant heat, hot plate) sensitivity was unaffected by virus to downregulate the neuronal TLR9 *p* > 0.05. Multiple comparisons using two-way ANOVA are the statistical approach.

In the CFA-treated mice, downregulation of neuronal TLR9 increased the mechanical paw withdrawal threshold as compared to the group with empty vector virus injection (Figure 5A), which indicated that neuronal TLR9 downregulation in the spinal cord prevented CFA-induced inflammatory pain.

Previous studies indicated that TLR9 deficiency impacted motor and sensory behaviors (Khariv et al., 2013). Thus, we detected whether downregulation of TLR9 in spinal neurons would affect sensory and motor behaviors. Downregulation of TLR9 in spinal neurons had no effect on locomotor activity (the index of the number of rearings and the traveled distance in Open Field Test, latency to fall in Rotarod test), mechanical sensitivity (von Frey test), and thermal sensitivity (Radiant heat and Hot plate) (*p* > 0.05, Figures 5B–G).

## Discussion

Our study for the first time demonstrates that TLR9 antagonist via intrathecal administration or downregulation of neuronal TLR9 attenuates CFA-induced inflammatory pain in mice. Thus, TLR9, particularly neuronal TLR9, may serve as a potential therapeutic target against inflammatory pain.

According to previous reports, there are several TLRs expressing in spinal cord including TLR2, TLR3, TLR4, TLR8, and TLR9 (Kigerl and Popovich, 2009; Heiman et al., 2014). The role of TLRs in all kinds of pain has been extensively studied (Liu et al., 2012; Ji, 2015). For instance, there are deferred and decreased mechanical allodynia and heat hyperalgesia post L5 spinal nerve transection in TLR2 knock-out mice (Kim et al., 2007). TLR4 ablation or inhibition by siRNA or antagonist attenuates arthritic pain (Christianson et al., 2011) and bone cancer pain (Wu et al., 2010). Taken together, these findings imply that TLRs have distinct and non-redundant functions. Earlier studies reveal that when intrathecally administrated, TLR9 agonist induces an inflammatory response in the spinal cord of intact mice (Li et al., 2019). TLR9 antagonist blocks tumor-induced pain sensitivity (Qi et al., 2011). In this study, the expression of TLR9 was increased by CFA-injection, and inhibition or downregulation of TLR9 alleviated CFA-induced mechanical pain, which supports the idea that TLR9 plays a detrimental role in CFA-induced inflammatory pain. TLR9 antagonists have been shown to preserve injured proximal axons, control glial scarring, and minimize detrimental inflammation through the action of ODN 2088 (Li et al., 2019). Recently, Dynavax Technologies (DT) has designed immunoregulatory oligonucleotides with unique inhibitory sequences for TLR9 that suppress autoimmune and inflammatory diseases (Patra and Choi, 2016). All the evidences above imply that TLR9 antagonist can be effective therapies for TLR9-mediated inflammatory disorders.

TLR9 has been lately confirmed to be expressed in different cell types in CNS. In diabetic encephalopathy, Sun et al. found a drastic increase for TLR9 expression in neurons cultivated in high-glucose media (Sun et al., 2017; Zhao et al., 2018). Our study indicated that TLR9 was mainly distributed in neuron in CFA-induced inflammation, and that neuronal TRL9 contributed to CFA-induced inflammatory pain. The Pearson’s *R* value were 0.37 ± 0.11 between TLR9-IR and NeuN-IR, representing the positive linear specific relevance between TLR9 and NeuN (R = [0, 1]). However, the proportion of TLR9 + in GFAP + were 0.08 ± 0.06, while the Pearson’s *R* value were 0.11 ± 0.01, representing weak specific relevance of TLR9-IR to GFAP-IR in CFA-induced inflammation. These results suggested that although we detected few TLR9-IR colocalized with GFAP-IR, there was still less relevance of TLR9 to GFAP in CFA-induced inflammatory pain. Even though TLR9 in astrocytes was found to be involved in SCI (spinal cord injury), and TLR9 antagonist could selectively affect astroglial glutamate transporters (Pallottie et al., 2018), whether astroglial TLR9 is involved in CFA-induced inflammatory pain still needs to be confirmed.

It has been reported that TLR9 deficient (TLR9^–/–^) mice displayed hypersensitivity to thermal stimuli and enhanced motor-responsivity compared to wide type mice. This indicated that TLR9 was probably of essential part for the development of sensory and motor behaviors (Khariv et al., 2013). In this study, downregulated neuronal TLR9 by AAV vector did not affect locomotor activity or sensitivity to mechanical and thermal stimulations. It should be noted that, in this study, the virus injection was performed on the mice aged 5–6 weeks and that we only downregulated TLR9 in neuron. This is quite different from the TLR9 deficient mice. The neural development of mice might be close to maturity when they are 5–6 weeks old, and further downregulation of TLR9 at this age is not enough to affect sensory-motor ability. On the other hand, despite the downregulation of TLR9 in some neurons, TLR9 in other cells has already met the normal neural development and function needs. Therefore, this experiment is different from that of TLR9 knockout mice. The downregulation of neuronal TLR9 did not show a hyper-responsive sensory and motor phenotype.

It is plausible that CpG ODN 2088 could confer neuronal protection by interfering with the binding or function of DAMPs that activate TLR9. However, there have been only a few identified TLR9 endogenous activators up to now. Fortunately, a number of host TLR9 stimulants have been discovered in the domain of sterile inflammation, such as mitochondrial DNA (Oka et al., 2012) and DNA-high-mobility group protein B1 (HMGB1) complex (Ivanov et al., 2007; Tian et al., 2007). Whereas the identification of DAMPs in CFA-affected spinal cord is beyond the scope of today’s investigation, future studies are in need to unravel such ligands.

## Data availability statement

The datasets used or analyzed during the current study are available from the corresponding author on reasonable request.

## Ethics statement

The animal study was reviewed and approved by local Ethical Committee at Fudan University of Basic Medical Sciences, People’s Republic of China (Agreement No. 20140 226-087).

## Author contributions

YC carried out the major part of the study and drafted the manuscript. HC and X-CL performed part of the study. W-LM and Y-XC revised the manuscript. Y-QW and Q-LM-Y conceived, designed the study, and revised the manuscript. All authors contributed to the article and approved the submitted version.
